# House Parties: An Innovative Model for Outreach and Community-Based Health Education

**DOI:** 10.1007/s10995-017-2378-9

**Published:** 2017-11-22

**Authors:** Timika Anderson-Reeves, Jacqueline Goodman, Brian Bragg, Chelsey Leruth

**Affiliations:** Access Community Health Network, 600 W Fulton, Suite 200, Chicago, IL 60661 USA

**Keywords:** Health care, Community health workers, Education, Social determinants of health

## Abstract

*Purpose* To connect low resource communities to innovative services that address gaps in health access and knowledge. *Description* We describe the house party model, as a community-based workshop approach to health education developed by the Westside Healthy Start program (WHS) in Chicago, Illinois. Key elements of the WHS house party model include use of community health workers as facilitators, collaboration with participants and community-based organizations, referrals to health care and social services, and engagement strategies such as interactive activities, personal stories, and discussion. *Assessment* In 2014 and 2015, WHS completed 23 house parties with 271 participants, delivering education on relevant maternal and child health (MCH) topics. Participants demonstrated improvements in knowledge of several health-related areas. About half of participants were able to identify causes or signs of preterm labor prior to the house party, compared to over 80% after. In addition, 94% of participants rated the house party workshops “excellent” or “good”. *Conclusion* House parties are a promising strategy for increasing knowledge about MCH topics and linking hard-to-reach populations to resources in the community.

## Significance

*What is already known on this subject*? Health education and access to care are important social determinants of health. Low resource communities served by the national Healthy Start program are in particular need of services to address gaps in health access and knowledge.

*What does this study add*? The house party model for community-based health education is a promising strategy for increasing knowledge about MCH topics and providing links to health care and social services for diverse, hard-to-reach populations.

## Purpose

The National Healthy Start (NHS) program model seeks to reduce infant mortality in the highest risk communities in the United States using five approaches: improve women’s health, promote quality services, strengthen family resilience, achieve collective impact, and increase accountability through quality improvement, performance monitoring, and evaluation (Maternal and Child Health Bureau, 2016). In 1997, Access Community Health Network launched the Westside Healthy Start program (WHS) to improve perinatal health outcomes by providing maternal and child health (MCH) services to high-risk, predominantly African-American women and children residing in Chicago. Currently, we serve approximately 800 families annually who experience some of the highest poverty rates in the nation. Our WHS target area also has an infant mortality rate of 14.1 per 1000 live births, which is more than double the national figure (Illinois Department of Public Health, 2014).

According to the Centers for Disease Control and Prevention (CDC), to address social determinants of health that contribute to infant mortality, there is a need to improve the delivery of quality health education and improve access to health care services (Centers for Disease Control and Prevention, 2017). Understanding the link between health disparities and infant mortality, the engagement of community health workers (CHW) has shown to be an effective strategy to bridge the gap between community members and health care services (Brown et al. 2011; Ingram et al. 2016). Hence, the need for an innovative model of outreach and community-based health education approach that utilizes CHWs, to connect low-resource communities to innovative services that strengthen the gaps in accessing health care services and delivery of quality-based health education. CHWs are laypersons who reside within the community, provide health education, and offer trusted resources to community residents. CHWs also serve as part of a multidisciplinary team within systems of care to identify and raise awareness of MCH services (Sudarsan et al. 2011). Based on this team approach, the development of a “House Party” model allowed CHWs to reach WHS participants and community members by using their trusted networks and support systems to engage in the discussion and facilitation of social problems that continue to create barriers to accessing care.

## Description

The house party model is a unique strategy for outreach and delivery of community health education to promote access to care, increase knowledge on MCH topics, and link individuals to social service resources in a fun and engaging workshop- or “house party”-format. CHWs facilitated house parties for small groups ranging from eight to ten attendees, including pregnant and interconceptional program participants and community residents. Events were held at the participant’s home or at a community-based organization’s meeting place. By engaging with program participants and community members in their own environments, CHWs created a safe space to comfortably discuss sensitive issues and strengthen relationships between WHS participants, friends, and family members (including grandmothers and male partners). Workshop attendees were educated on topics that gave them the knowledge and skills to support mothers during and after pregnancy, the child’s first two years of development, and throughout the lifespan. House party workshops also served as a venue to display the importance of having strong ties with community-based organizations that empowered attendees to gain additional knowledge and resources.

### House Party Model Development

The WHS program learned of the house party concept, seen as an urban ritual with the intention for community members to gather and share information, at a National Healthy Start Association annual conference and brought the idea to Chicago. The development of the house party model involved designing a robust health education curriculum that included staff facilitation training and observations completed by the program supervisor to ensure material was relevant to the needs of the community. Program administrators, a master’s level public health intern, and an evaluator worked together to find evidence-based educational materials, from sources including March of Dimes, CDC, and the United States Department of Agriculture, that became the basis for the key health messaging and interactive activities at the house party. The goal in designing the house party curriculum was to convey key health educational messages concerning the importance of early access to prenatal care and having a health care provider located within a patient-centered medical home (PCMH). The house party concept is innovative and aligns with the PCMH model, which emphasizes relationship-based primary care that addresses the needs of the whole person (Agency for Healthcare Research and Quality, n.d.). Furthermore, the house party curriculum included topics on healthy pregnancy, family planning, and stress management. In addition to training on the curriculum, CHWs received training on facilitation techniques that included cultural awareness and diversity, adult learning styles, and adaptation of material to accommodate low literacy levels. Once trained, CHWs piloted house party workshops with WHS participants and community residents. Evaluation staff observed workshops to develop tools to assess participant satisfaction and change in participant knowledge, attitudes, and skills for each workshop.

### Recruitment

The WHS program found success in a two-step strategy for scheduling and recruiting for each house party workshop. First, WHS CHWs distributed fliers, with staff contact information, to program participants and community-based organizations informing them of free health education workshops on MCH topics. Based on their needs and interest area, participants or community organizations contacted WHS staff to schedule a house party workshop on the topic of their choice. This participant or organization served as the “host” of the house party, who is often recognized as a leader in the community, and worked in tandem with the CHW facilitator to recruit attendees for the house party. This process allowed the host to invite “guests” who they thought might benefit from receiving health educational messages and learning how to support women during preconception, pregnancy, and interconception phases of the lifespan.

To ensure there was an appropriate amount of presentation materials and healthy refreshments at the workshop, the host notified the CHW of the number of confirmed attendees within 48 hours of the event. Program participant hosts also received a $10 gift card as a thank you for hosting the workshop at their home and recruiting their friends and family to attend. However, community-based organizations were not eligible for the gift card. Often, CHWs were able to schedule subsequent house party workshops with the same host or other attendees based on the rapport developed during the house party.

### Workshop Format and Facilitation

House party workshops included a variety of elements to engage attendees. CHWs used up to $25 to purchase healthy refreshments from a local grocery store. Healthy refreshments helped CHWs foster a warm environment to facilitate engagement, and lessened the burden on the host to buy food for workshop attendees. House party workshops were scheduled to be no more than 90 minutes but were sometimes extended based on the needs of the attendees. During the house party workshop, the CHW promoted health education through interactive activities- such as pregnancy bingo and family planning Jeopardy- shared personal stories, and distributed prizes. This helped to create a lively atmosphere and build trust and credibility between attendees. Participants learned directly from one another in a comfortable, trusting environment that allowed commonly held beliefs or myths about the topics to be discussed and dispelled. At the end of the workshop, attendees received an informational “fun facts” sheet to take home, which highlights areas of importance concerning the house party topic. For example, the healthy pregnancy fun facts included “breastfeeding has many more health benefits than formula for you and your baby, and it’s completely free.” Workshop attendees in need of healthcare or social services received referrals to organizations within the WHS target area and neighboring communities. Providing education and referrals for the participants also empowered them to share their new knowledge with other community members.

## Methods

At the beginning and end of each house party workshop, attendees voluntarily completed a brief questionnaire or “survey” that requested responses on the selected topic with a field to input their date of birth to match pretest and posttest. The questionnaire also included closed and open-ended questions measuring health knowledge, attitudes, and self-efficacy related to the workshop’s topic. Response options were multiple choice or on a Likert Scale. Additional open-ended questions on the posttest measured general satisfaction with the workshop. Completed questionnaires were converted into electronic format using ExpertScan software, and evaluation staff manually entered open-ended responses. Additionally, CHWs recorded aggregate data on the location of the house party, number of workshop attendees, topic, and completion of evaluation tools in an online project management spreadsheet.

## Results

In 2014 and 2015, the WHS staff completed 23 house parties with 271 participants on the topics of healthy pregnancy, family planning, and stress. Questionnaires were completed at 16 of the house parties. During this period, 74% of these house parties took place in the WHS target area.

### Short-Term Changes in Health-Related Outcomes

Participants reported improvements in most health-related knowledge, as shown in Table 1. Notably, > 75% of participants noted that healthy pregnancy, family planning, and stress were important to them before the house party. Additionally, only about half of participants were able to identify causes and signs of preterm labor prior to the house party compared to > 80% at post-test.

**Table 1:** Health-related knowledge

House party topic	Baseline	Follow-up
*Healthy pregnancy*		
Topic somewhat or very important (attitude)	76% (n = 51/67)	88% (n = 50/57)
Somewhat or strongly agree it’s important to see doctor as soon as possible when pregnant (attitude)	87% (n = 58/67)	97% (n = 57/59)
Know STDs can cause premature labor (knowledge)	49% (n = 29/59)	84% (n = 46/55)
Know water leaking from vagina is a symptom of premature labor (knowledge)	62% (n = 40/65)	86% (n = 48/56)
*Stress*		
Topic somewhat or very important (attitude)	94% (n = 50/53)	93% (n = 51/55)
Feel somewhat or very stressed (health assessment)	53% (n = 28/53)	22% (n = 12/55)
Somewhat or strongly agree it’s easy to recognize stressors (self-efficacy)	73% (n = 38/52)	65% (n = 35/54)
Know exercise is a healthy way to relieve stress (knowledge)	62% (n = 28/45)	84% (n = 42/50)
*Family planning*		
Topic somewhat or very important (attitude)	89% (n = 31/35)	97% (n = 34/35)
Know you can get pregnant at first sexual encounter (knowledge)	87% (n = 31/35)	94% (n = 33/35)
Know condoms prevent pregnancy and STDs (knowledge)	97% (n = 31/32)	100% (n = 30/30)
Somewhat or very likely to use birth control at next sex (intention)	89% (n = 32/36)	89% (n = 31/35)

Sample sizes differ between baseline and follow-up assessments due to time and participant constraints

### Satisfaction with House Parties

**Fig. 1:**
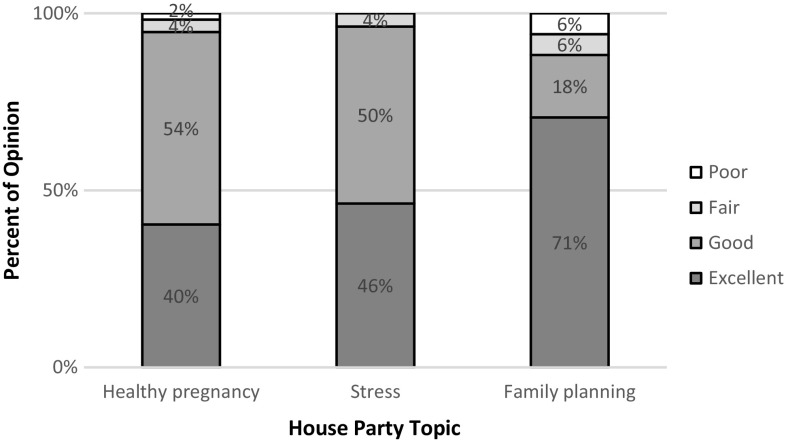
What is your overall opinion of the house party?

Following the house party, most participants had a favorable opinion of the workshop, as seen in Fig. 1.

**Fig. 2:**
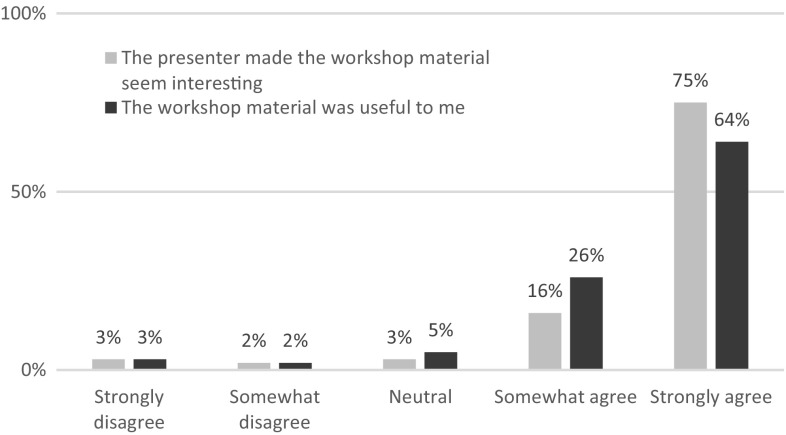
Interest in and usefulness of the material

Figure 2 shows that most participants found that the presenter made the material seem interesting, and that the material discussed was useful.

The surveys allowed participants to provide comments about what they particularly liked or disliked about the house party workshop. Of those who provided comments, 30% (30/101) of attendees expressed an overall satisfaction with the house party, 37% (n = 37) enjoyed the game or activity, and 26% (n = 26) noted that they learned something. Minimal dissatisfaction was noted; 3% (n = 3) found some of the material to be displeasing, and 2% (n = 2) suggested areas for improvement, including better visual materials.

## Conclusion

House parties are a promising strategy for increasing knowledge about MCH topics and providing links to health care and social services for diverse, hard-to-reach populations. This model also provides a creative approach to implement all five NHS program strategies, which include improving women’s health (by promoting access to care), promoting quality services (by encouraging health practices such as safe sleep, reducing stress, and family planning), strengthening family resilience (by addressing stress and educating women’s friends and family members), achieving collective impact (by partnering with community-based organizations to extend the reach of WHS services to the wider community), and increasing accountability through quality improvement, performance monitoring, and evaluation (by collecting, analyzing, and disseminating evaluation findings).

### Limitations

Our evaluation strategy has some limitations. CHWs rely on the ‘hosts’ to recruit house party workshop attendees who may or may not need health education related to MCH topics.

Participants who “self-select” to attend the house parties may be more informed about MCH topics or more invested in learning than other community members who do not attend. However, data from pretest surveys indicates that the house party topics are important to participants and baseline knowledge on the healthy pregnancy items was low, indicating that house parties are relevant to participants. Since participants are not randomly selected, the ability to generalize these results to the broader community is limited. Due to time and participant constraints, questionnaires are not always completed introducing the possibility of non-response bias. Participants sometimes leave the workshops early or arrive late, so data may include responses from somewhat different groups of respondents for the pretest versus post-test. Our findings are limited to immediate post-test results, so we cannot assess knowledge retention, change in attitudes over time, or any effect on health behavior.

### Acceptability and Relevance

Evaluation results indicate that community participants are pleased with the house party model. They report very favorable opinions about the workshops overall, facilitation style, and usefulness of the content. Organizations seeking to replicate this model should ensure that workshops include interactive games, group discussion, food, and prizes since these are the aspects of the house parties participants identified as most enjoyable.

In addition, pretest results indicate that the MCH topics covered in our house parties are very relevant for participants. Low levels of knowledge about these topics on the pretest also demonstrate that WHS house parties reach populations in need of education.

### Healthy Pregnancy

Overall, our healthy pregnancy session was the most successful, possibly due to facilitators’ competency and expertise with this topic. Improvements in knowledge about causes of preterm labor indicated success in meeting a large need for education on this topic. This improvement in pregnancy knowledge is especially relevant given the high rates of prematurity among our target population.

### Stress Management

Stress is an important mediator of overall health and has been shown to contribute to race/ethnic disparities in birth outcomes (Dole et al. 2003; Lu and Halfon 2003). One stress management workshop is not likely to have a measurable impact on overall health, but this session aims to provide participants with knowledge and tools they can use moving forward. Most results for this topic were favorable. Participants indicated lower levels of self-reported stress after the house party, and more were able to identify a healthy way to relieve stress. Contrary to the session’s objectives, fewer participants reported confidence in their ability to recognize stressors. It is possible that participants over-estimated their abilities on the pretest, and then judged their abilities more critically after learning more about different types of stressors and stress responses. This phenomena is common with pre- and post-skill assessments, so it may be more reliable to collect baseline data on this item retrospectively on the post-test (Pratt et al. 2000).

### Family Planning

Family planning is an important strategy to prevent unintended pregnancy, improve birth spacing, and foster educational attainment and financial security (Sonfield 2013). Although most results for this topic were favorable, nearly all participants already knew that condoms protect against pregnancy and sexually transmitted infections, indicating that this message is not relevant for the adult population served through our events. Moving forward, we plan to revise the curriculum for this session and select learning objectives, health messages, and evaluation items that are more appropriate for the target population.

### Future Expansion

Additional presentations were developed based on interest from program participants, including immunizations, sudden infant death syndrome (SIDS), and advocacy. Evaluation forms are currently in development for these topics.
